# Acute small bowel obstruction as a result of a Meckel's diverticulum encircling the terminal ileum: A case report

**DOI:** 10.1186/1752-1947-1-8

**Published:** 2007-03-23

**Authors:** Avnesh S Thakor, Siong S Liau, Dermot C o'Riordan

**Affiliations:** 1 Department of Surgery, West Suffolk Hospital, Bury St. Edmunds, IP33 2QZ, UK

## Abstract

**Background:**

In the developed world, small bowel obstruction accounts for 20% of all acute surgical admissions. The aetiology for majority of these cases includes postoperative adhesions and herniae. However, a relatively uncommon cause is a Meckel's diverticulum. Although this diagnosis is primarily reported in the adolescent population, it should also be considered in adults.

**Case Presentation:**

In the present report, we present a rare case where a fit and healthy 74-year-old gentleman, with no previous history of abdominal surgery, presented with the cardinal symptoms and signs of small bowel obstruction as the result of a Meckel's diverticulum encircling his terminal ileum. Initial investigations included a supine abdominal x-ray showing dilated loops of small bowel and computerised tomographic imaging of the abdomen, which revealed a stricture in the terminal ileum of unknown aetiology. At laparotomy, multiple loops of distended small bowel were seen from the duodeno-jeujenal junction to the terminal ileum, which was encircled by a Meckel's diverticulum. The Meckel's diverticulum was then divided to release the obstruction, mobilised and subsequently removed. Finally, the small bowel contents were decompressed into the stomach and the nasogastric tube aspirated, before returning the loops of bowel into the abdomen in sequence. The patient made a good postoperative recovery and was discharged home 5 days later.

**Conclusion:**

This report highlights the importance of considering a Meckel's diverticulum as a cause of small bowel obstruction in individuals from all age groups and especially in a person with no previous abdominal pathology or surgery.

## Case Presentation

### Background

In the developed world, small bowl obstruction accounts for 20% of all acute surgical admissions. The aetiology of small bowel obstruction includes several pathological factors, with the most common cause being postoperative adhesions followed by herniae [1]. However, in patients who present with the symptoms and signs of bowel obstruction and who have had no previous abdominal surgery, or any detectable herniae on physical examination, other causes such as a Meckel's diverticulum should be considered.

A Meckel's diverticulum is a congenital pouch on the wall of the distal ileum, usually about 2 inches from the ileocecal valve. It represents a vestigial remnant of the omphalomesenteric duct and occurs in approximately 2% of the population, found twice as frequently in males as females. Of those individuals who have a Meckel's diverticulum, only 2% are symptomatic and they tend to be typically below the age of two, thereby accounting for why this congenital gastrointestinal anomaly is comparatively better studied in adolescents compared to in adults.

The main complications caused by a Meckel's diverticulum, include intersusseption and volvulus in adolescents and acute bleeding in adults [2]. However, there are cases reported in the literature of a Meckel's diverticulum causing small bowel obstruction [3-6], but this predominantly occurs in adolescents where the bowel lumen is narrower and the intra-abdominal contents are more closely packed together.

Here, we present a case of a Meckel's diverticulum causing acute small bowel obstruction in a 74-year-old gentleman as a result of it encircling, and thus constricting, the terminal ileum. To the authors' knowledge, and from an extensive review of the literature, such an unusual presentation of a Meckel's diverticulum has not been previously reported.

## Case Report

A fit and healthy 74-year-old gentleman presented to the accident and emergency department at the West Suffolk Hospital with a 3-day history of abdominal pain, vomiting, absolute constipation and abdominal distension. The abdominal pain initially started as a dull generalised discomfort, but later became colicky in nature with a subjective severity of 7/10. There were no other abdominal or genitourinary symptoms. The patient had an unremarkable past surgical history, with no prior abdominal surgery, and a past medical history of only hypercholesterolaemia.

On examination, positive findings included marked abdominal distension, generalised abdominal tenderness, tinkling bowel sounds and soft stools high in the rectum. Important negative findings included no herniae and no signs of peritonism.

**Figure 1:**
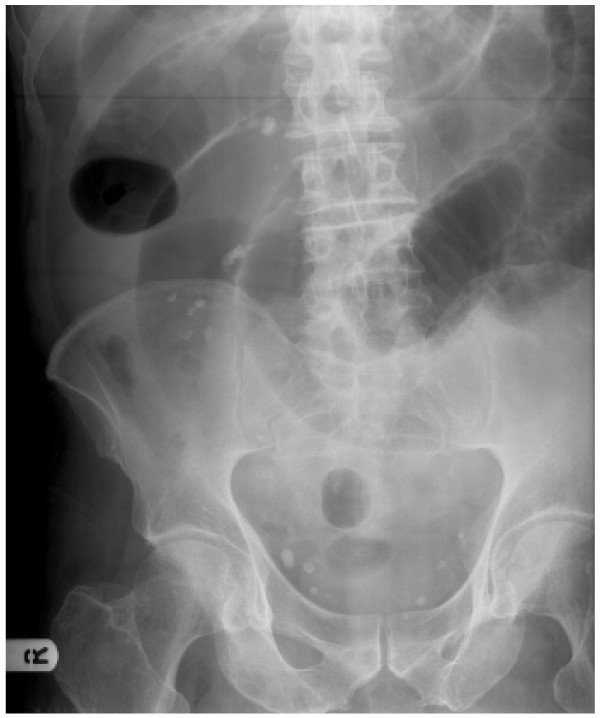
This x-ray shows multiple loops of dilated small bowel.

**Figure 2:**
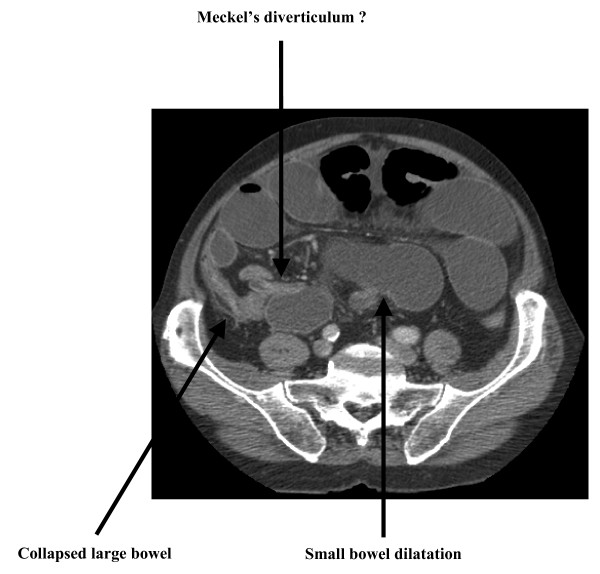
**Preoperative helical computed tomography transverse scan of the abdomen**. This image shows small bowel obstruction as a result of a stricture in the terminal ileum.

Initial management of the patient involved intravenous fluid resuscitation, nasogastric tube insertion, catheterisation, routine bloods and erect chest and supine abdominal x-rays. Significant elevations in blood concentrations of urea, creatinine and C-reactive protein were noted, with dilated loops of small bowel (Fig. 1) and no free air under either diaphragm on x-ray. Over the next 12 hours, the patient's vital signs remained stable and his condition did not deteriorate further. To identify the cause of the small bowel obstruction, computerised tomographic imaging of the abdomen with oral contrast was performed which revealed dilated loops of small bowel with a stricture in the ileum and collapse of the distal ileum and large bowel (Fig. 2). As the aetiology of the stricture remained unidentified, the decision was made to perform a diagnostic laparotomy and manage the patient accordingly.

Following general anaesthesia, a midline laparotomy was performed on the patient. On entering the peritoneal cavity, gross distension of the small bowel and collapse of the large bowel was identified. The small bowel was subsequently delivered carefully and examined. Loops of distended small bowel were identified extending proximally from the duodeno-jejunal junction to the distal ileum. At approximately 10 cm from the ileo-caecal valve, there was a long tubular structure encircling and obstructing the terminal ileum, which proved to be a Meckel's diverticulum. The base of the Meckel's diverticulum arose approximately 40 cm proximal from the ileo-caecal valve. The encircling Meckel's diverticulum did not appear inflamed or thickened and was divided at the base using a linear stapler (TLC55, Ethicon) to release the obstruction. Care was taken not to compromise the lumen of the ileum. The tip of the diverticulum was then dissected off the terminal ileum and the anastomosis over sewn with continuous 3/0 sutures. The small bowel was then decompressed and the content milked gently into the stomach before being aspirated via the nasogastric tube. The loops of bowel were then returned into the abdomen in sequence. Closure of the abdomen was performed using loop sutures. Following this, the patient made a good postoperative recovery and was discharged home 5 days later.

## Discussion

The management of any acute surgical abdomen, including acute bowel obstruction, follows 4 stages: (I) formation of an initial diagnosis, (II) confirmation of a diagnosis, (III) confirmation of the aetiology underlying the diagnosis and (IV) surgical intervention to treat the emergency.

A diagnosis of acute bowel obstruction is made initially on clinical judgement based on the history and physical examination of the patient. The cardinal symptoms and signs are colicky abdominal pain, vomiting, absolute constipation and abdominal distension, all of which were present in this patient.

Confirmation of bowel obstruction is then usually made with a plain supine abdominal x-ray. This simple and easily performed test provides the surgeon with several useful pieces of information, including whether there is small and/or large bowel obstruction and the degree of obstruction. In the present case, markedly dilated loops of small bowel with no visible loops of large bowel were seen on the abdominal x-ray (Fig. 1), thus indicating acute small bowel obstruction.

Having established and confirmed a diagnosis of small bowel obstruction, the next goal is to identify the aetiology underlying the obstruction. The two most common causes of small bowel obstruction in the developed world are postoperative adhesions and herniae [1]. However, this patient had no previous abdominal surgery and no herniae on physical examination, therefore making both these causes unlikely. Hence, it was decided to image his abdomen with a computed tomography scan with oral contrast. The result of this revealed a stricture in the terminal ileum, with dilatation of the small bowel proximal and collapse of the large bowel distal to the stricture (Fig. 2). However the aetiology of the stricture, and therefore the cause of the small bowel obstruction, remained unidentified.

Based on these findings, and the absence of clinical improvement whilst on IV fluids and nasogastric tube aspiration, surgery was therefore indicated. However, the surgical approach to acute bowel obstruction of unknown aetiology remains controversial. While some surgeons advocate laparoscopic intervention due to its minimally invasive approach and shorter patient hospitalisation [7], others favour an open laparotomy due to the larger surgical space and lower incidence of bowel injury. Further evidence to support the latter approach comes from Kirshtein and colleagues who reviewed 65 cases of acute bowel obstruction that were initially managed laparoscopically [8]. In that study, although laparoscopy was shown to have a diagnostic accuracy of 96.9%, a significant number of cases still required conversion for their subsequent management. Based on the above literature and the pervious experience of this surgical team, it was therefore decided that this patient should undergo an open laparotomy.

**Figure 3:**
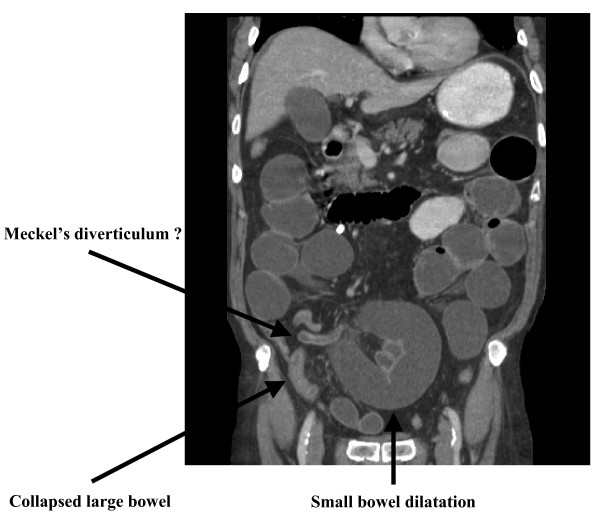
**Reconstructed computed tomography coronal scan of the abdomen**. This image shows small bowel obstruction as a result of a stricture in the terminal ileum. A postoperative review suggested a Meckel's diverticulum could be described.

At laparotomy, an unusually long Meckel's diverticulum was found, which had managed to entirely wrap itself around the terminal ileum thereby forming an internal hernial orifice in which the bowel had become incarcerated and subsequently obstructed. What makes this case exceptionally unusual is that the Meckel's diverticulum was not thickened or inflamed. This is in contrast to the other cases previously reported in the literature, where an internal hernial orifice was created by the Meckel's as the result of adhesions or bands between an inflammatory end of the diverticulum and either the surrounding mesentery [9] or the neighbouring appendix [3]. On retrospective analysis of both the preoperative helical (Fig. 2) and reconstructed computed tomography (Fig. 3) scans, the Meckel's diverticulum could now be identified as being the cause for the stricture of the terminal ileum and therefore the cause of the small bowel obstruction.

## Conclusion

This report therefore highlights the importance of considering a Meckel's diverticulum as a cause of small bowel obstruction in individuals from all age groups and especially in a person with no previous abdominal pathology or surgery.

## Competing interests

The author(s) declare that they have no competing interests.

## Authors' contributions

All authors have read and approved the final manuscript.

**AST **(Surgical House Officer): Involved in the conception of the report, literature review, manuscript preparation, manuscript editing and manuscript submission.

**SSL **(Surgical Registrar): Involved in the manuscript editing and manuscript review.

**DOR **(Consultant Surgeon): Involved in the manuscript editing and manuscript review.
